# Supplementary material for: Classification performance of administrative coding data for detection of invasive fungal infection in paediatric cancer patients
Source: PLoS One. 2020 Sep 9;15(9):e0238889. doi: 10.1371/journal.pone.0238889 (PMC7480858; doi:10.1371/journal.pone.0238889)
Supplement: S3 Table — (PDF) [file pone.0238889.s003.pdf]

Supporting Information

Table S3. ICD-10-AM coding conventions for invasive fungal infection, haematological malignancy and solid tumour neoplasms

Composite code definitions	Infection/cancer	ICD-10-AM code(s)
Invasive fungal infection		
Invasive candidiasis	Candidal endocarditis	B37.6
	Candidal meningitis	B37.5
	Candidal sepsis	B37.7
	Candidiasis of other sites	B37.88
Chromomycosis and phaeomycotic abscess	Chromomycosis, unspecified	B43.9
	Cutaneous chromomycosis	B43.0
	Other forms of chromomycosis	B43.8
	Phaeomycotic brain abscess	B43.1
	Subcutaneous phaeomycotic abscess and cyst	B43.2
Cryptococcosis	Cerebral cryptococcosis	B45.1
	Cryptococcosis, unspecified	B45.9
	Cutaneous cryptococcosis	B45.2
	Disseminated cryptococcosis	B45.7
	Osseous cryptococcosis	B45.3
	Other forms of cryptococcosis	B45.8
	Pulmonary cryptococcosis	B45.0
Invasive aspergillosis	Aspergillus, unspecified	B44.9
	Invasive pulmonary aspergillus	B44.0
	Disseminated aspergillosis	B44.7
	Other forms of aspergillosis	B44.8
	Pulmonary aspergillosis, other	B44.1
	Tonsillar aspergillosis	B44.2
Mucormycosis	Cutaneous mucormycosis	B46.3
	Disseminated mucormycosis	B46.4
	Gastrointestinal mucormycosis	B46.2
	Mucormycosis, unspecified	B46.5
	Other zygomycoses	B46.5
	Pulmonary mucormycosis	B46.0
	Rhinocerebral mucormycosis	B46.1
	Zygomycosis, unspecified	B46.9
Mycetoma	Actinomycetoma	B47.1
	Eumycetoma	B47.0
	Mycetoma, unspecified	B47.9
Pneumocystosis	Pneumocystosis	B50
Sporotrichosis	Disseminated sporotrichosis	B42.7
	Lymphocutaneous sporotrichosis	B42.1
	Other forms of sporotrichosis	B42.8

Composite code definitions	Infection/cancer	ICD-10-AM code(s)
	Pulmonary sporotrichosis	B42.0
	Sporotrichosis, unspecified	B42.9
Invasive fungal infection, other	Allescheriasis	B48.2
	Geotrichosis	B48.3
	Lobomycosis	B48.0
	Opportunistic mycoses	B48.7
	Other specified mycoses	B48.8
	Penicillosis	B48.4
	Rhinosporidiosis	B48.1
Unspecified mycosis	Unspecified mycosis	B49
Cancer		
Haematological malignancy	Acute lymphoblastic leukaemia	C910 (0/1), C918 (0/1)
	Acute myeloid leukaemia	C920 (0/1), C925 (0/1), C926 (0/1), C928 (0/1), C940 (0/1), C942 (0/1), C930 (0/1), C924 (0/1), C943 (0/1), C944 (0/1), C923 (0/1)
	Chronic lymphocytic leukaemia	C911 (0/1), C914 (0/1), C916 (0/1)
	Chronic myeloid leukaemia	C921 (0/1), C922 (0/1)
	Hodgkin lymphoma	C810, C811, C812, C813, C814, C817, C819
	Non-Hodgkin lymphoma	C820, C821, C822, C823, C824, C825, C826, C827, C829, C830, C831, C833, C835, C837, C838, C839, C840, C841, C844, C846, C847, C851, C852, C957, C859, C880 (0/1), C884 (0/1), C848, C849, C860, C861, C862, C863, C865, C866, C833 (0/1)
	Multiple myeloma	C900 (0/1), C902 (0/1), C903 (0/1)
	Myelodysplastic syndrome	D46, C946 (0/1)
	Haematological malignancy, other	C845, C882 (0/1), C887 (0/1), C889 (0/1), C901 (0/1), C915 (0/1), C917 (0/1), C919 (0/1), C927 (0/1), C929 (0/1), C931 (0/1), C937 (0/1), C939 (0/1), C947 (0/1), C950 (0/1), C951 (0/1), C957 (0/1), C959 (0/1), C960, C962, C964, C965, C966,

Composite code definitions	Infection/cancer	ICD-10-AM code(s)
		C967, C968, C969, C941 (0/1), C913 (0/1), C918 (0/1)
Solid tumour neoplasm	Solid tumour neoplasm	C00 – C76, C80
	Neuroblastoma	C47, C61, C64, C71, C74

Abbreviations: ICD-10-AM, *International Statistical Classification of Health Interventions and Related Problems, Tenth Revision, Australian Modification*
