# Supplementary material for: Classification performance of administrative coding data for detection of invasive fungal infection in paediatric cancer patients
Source: PLoS One. 2020 Sep 9;15(9):e0238889. doi: 10.1371/journal.pone.0238889 (PMC7480858; doi:10.1371/journal.pone.0238889)
Supplement: S4 Table — (PDF) [file pone.0238889.s004.pdf]

Supporting Information

Table S4. Australian Classification for Health Intervention codes denoting allogeneic and autologous haematopoietic stem cell transplantations

Composite code definitions	Procedure	ACHI code(s)
Haematopoietic stem cell transplantation		
Allogeneic, matched related donor	Allogeneic bone marrow or stem cell transplantation, matched related donor, with *in vitro* processing	13706-06 [802]
	Allogeneic bone marrow or stem cell transplantation, matched related donor, without *in vitro* processing	13706-00 [802]
Allogeneic, other donor	Allogeneic bone marrow or stem cell transplantation, other donor, with *in vitro* processing	13706-09 [802]
	Allogeneic bone marrow or stem cell transplantation, other donor, without *in vitro* processing	13706-10 [802]
Autologous	Autologous bone marrow or stem cell transplantation, with *in vitro* processing	13706-07 [802]
	Autologous bone marrow or stem cell transplantation, without *in vitro* processing	13706-08 [802]

Abbreviations: ACHI, *Australian Classification of Health Interventions*
