# Supplementary material for: Classification performance of administrative coding data for detection of invasive fungal infection in paediatric cancer patients
Source: PLoS One. 2020 Sep 9;15(9):e0238889. doi: 10.1371/journal.pone.0238889 (PMC7480858; doi:10.1371/journal.pone.0238889)
Supplement: S6 Table — (PDF) [file pone.0238889.s006.pdf]

Supporting Information

Table S6. Performance (in Percent) of administrative coding data for case detection of modified possible and treated for invasive fungal infection classifications

Invasive fungal infection	All cancers (N=1,671)			Acute lymphoblastic leukaemia (N=516)			Acute myeloid leukaemia (N=110)			Neuroblastoma (N=397)		
	Sensitivity [95% CI]	PPV [95% CI]	F_1	Sensitivity [95% CI]	PPV [95% CI]	F_1	Sensitivity [95% CI]	PPV [95% CI]	F_1	Sensitivity [95% CI]	PPV [95% CI]	F_1
**Modified possible EORTC/MSG (N=6)**												
All invasive fungal infection	17 [0.42-64]	1.47 [0.04-7.92]	2.71	50 [1.25-99]	2.50 [0.06-13]	4.77	0 [0-0]	0 [0-0]	0	0 [0-0]	0 [0-0]	0
**Treated for invasive fungal infection EORTC/MSG (N=5)**												
All invasive fungal infection	40 [5.27-85]	2.94 [0.36-10]	5.48	0 [0-0]	0 [0-0]	0	-	-	-	100 [2.5-100]	14 [0.36-58]	25

EORTC/MSG, European Organization for Research and Treatment of Cancer/Invasive Fungal Infections Cooperative Group and the National Institute of Allergy and Infectious Diseases Mycoses Study Group; PPV, positive predictive value
